# Nature-inspired Enzyme engineering and sustainable catalysis: biochemical clues from the world of plants and extremophiles

**DOI:** 10.3389/fbioe.2023.1229300

**Published:** 2023-06-20

**Authors:** Anwesha Chatterjee, Sonakshi Puri, Pankaj Kumar Sharma, P. R. Deepa, Shibasish Chowdhury

**Affiliations:** Department of Biological Sciences, Birla Institute of Technology and Science (BITS), Pilani, India

**Keywords:** enzyme engineering, plants and algae, extremozymes, enzymatic yield, sustainable catalysis

## Abstract

The use of enzymes to accelerate chemical reactions for the synthesis of industrially important products is rapidly gaining popularity. Biocatalysis is an environment-friendly approach as it not only uses non-toxic, biodegradable, and renewable raw materials but also helps to reduce waste generation. In this context, enzymes from organisms living in extreme conditions (extremozymes) have been studied extensively and used in industries (food and pharmaceutical), agriculture, and molecular biology, as they are adapted to catalyze reactions withstanding harsh environmental conditions. Enzyme engineering plays a key role in integrating the structure-function insights from reference enzymes and their utilization for developing improvised catalysts. It helps to transform the enzymes to enhance their activity, stability, substrates-specificity, and substrate-versatility by suitably modifying enzyme structure, thereby creating new variants of the enzyme with improved physical and chemical properties. Here, we have illustrated the relatively less-tapped potentials of plant enzymes in general and their sub-class of extremozymes for industrial applications. Plants are exposed to a wide range of abiotic and biotic stresses due to their sessile nature, for which they have developed various mechanisms, including the production of stress-response enzymes. While extremozymes from microorganisms have been extensively studied, there are clear indications that plants and algae also produce extremophilic enzymes as their survival strategy, which may find industrial applications. Typical plant enzymes, such as ascorbate peroxidase, papain, carbonic anhydrase, glycoside hydrolases and others have been examined in this review with respect to their stress-tolerant features and further improvement via enzyme engineering. Some rare instances of plant-derived enzymes that point to greater exploration for industrial use have also been presented here. The overall implication is to utilize biochemical clues from the plant-based enzymes for robust, efficient, and substrate/reaction conditions-versatile scaffolds or reference leads for enzyme engineering.

## 1 Introduction

With the rapid growth of industries, there is a constant need to assess their impact on the environment and develop ecologically friendly systems to bridge the gap between the increasing demands of modern society and the conservation of natural resources. This could be achieved by deploying sustainable alternatives in industries which can help us to face various environmental challenges like global warming and pollution. In this regard, the application of enzymes as catalysts for the synthesis of different products has proven to be more efficient and a much greener approach in contrast to its inorganic counterparts (Sheldon & Woodley, 2018). Another advantage of enzymes is that they are highly stereoselective which enables them to carry out a wide range of chemical transformations with unparalleled levels of reaction specificity (Kate et al., 2022).

Despite the manifold benefits of using enzymes, there are concerns over their relative stability owing to the extreme temperature and pH in the large-scale catalytic chambers in industries (Littlechild, 2015). Recent trends in biocatalysis, therefore, explore the application of extremozymes, that is enzymes employed by organisms thriving in extreme conditions (Herbert, 1992). Extremozymes are naturally tolerant to harsh environmental conditions such as high temperature and pressure, high salinity, water scarcity and extremes of pH. Since extremozymes are adapted to extreme physicochemical conditions, these are one of the most sought-after class of biocatalysts in industrial processes (Liszka et al., 2012). For instance, nuclease H, a halophilic enzyme isolated from *Micrococcus varians* subsp. *halophilus,* breaks down RNA at 60°C and 12% salt concentration, and is used for the production of a flavoring agent 5-guanylic acid in the food industry (Onishi et al., 1988). Another example is the L-aminoacylase enzyme derived from the thermophilic archaeon *Thermococcus litoralis,* which shows optimum activity at 85°C in Tris- HCl, pH 8 and has a broad substrate specificity (Toogood et al., 2002). This enzyme is used for the industrial synthesis of various L-amino acids and their analogs by several pharmaceutical companies.

One challenge with the implementation of enzymes from extremophilic organisms has been the limited success of recombinant protein expression in hosts (Liszka et al., 2012). This has been partly overcome by altering the host systems which however reduces the enzyme stability and catalytic activity. This is where the techniques of enzyme engineering can be exploited to overcome this problem and optimize enzymes for better yield, stability and efficiency. These techniques include the customization of amino acid sequences of an enzyme, by making changes in the covalent and hydrophobic or electrostatic interactions between the catalytic domains or the other flanking amino acid residues in the enzyme.

A conventional approach in improving enzyme yields for industrial scale-up processes has been to experiment with the unit operations towards process optimization. For instance, using biocalorimetry and metabolic profiling, a comparative analysis of an industrially important enzyme, Inulinase, was made between batch and fed-batch enzyme production in *Kluyveromyces marxianus* by optimizing substrate feeding rates (Leelaram et al., 2016). Such process engineering approaches may be complemented by modern biotechnological approaches that include enzyme engineering and recombinant DNA technology. To this end, plants provide ample opportunities to use them as bioreactors for enzyme production, or as inspiration for enzyme engineering. While microbes have been abundantly exploited for sourcing extremozymes, plants are emerging with complementary potential. This review aims to highlight different mechanisms for enzyme engineering to facilitate the generation of enzyme variants for industrial applications, and presents some special case studies of plant-based enzymes, including extremozymes.

## 2 Enzyme engineering

The prominence of enzymes in industries as biocatalysts is ever-increasing, owing to the advantages they have over traditional inorganic catalysts. However, when it comes to the use of native enzymes in industrial processes, there are a number of limitations that should be considered, especially when these enzymes are subjected to extreme conditions that are very different from their natural environment. These include lower catalytic efficiency, decreased tolerance towards organic solvents, lack of stability under high temperatures and pressure in large-scale industrial fermenters, which ultimately leads to poor product yield (Victorino da Silva Amatto et al., 2022). Hence, these wild-type enzymes need to be engineered to work under non-physiological conditions, with non-natural substrates, for the production of industrially important biochemicals.

Enzyme engineering has emerged as a potential technique to develop new robust enzymes and/or to enhance the existing enzyme properties which are crucial for industrial biotransformations. Engineering of enzymes can improve their catalytic activity, thermostability, substrate specificity or enhance their ability to work with a broader range of substrates (Steiner & Schwab, 2012; Mazurenko et al., 2019). The main aim of enzyme engineering is to optimize the activity of an enzyme by altering its amino acid sequence and thus, making it suitable to be used in industrial processes. The primary target in various approaches of enzyme engineering is gaining insights into the sequence and structure of the enzyme to be modified. This is based on the idea that the functional characteristics of an enzyme can be manipulated by leveraging information about the molecular complexity of its structure. During biocatalysis, the formation of the enzyme-substrate complex plays a major role in determining the kinetic efficiency of the enzyme. Therefore, while engineering enzymes it is usually deemed important to modulate changes in the amino acid residues present in the catalytic domain of the enzyme which, in most cases, are involved in the enzyme-substrate interactions. However, modifications in amino acids other than those found in the active site might also help to regulate enzyme-substrate interactions, by bringing about an overall alteration in the conformation of the enzyme and its functional improvisation (Wilding et al., 2019).

### 2.1 Methods for Enzyme engineering

**FIGURE 1:**
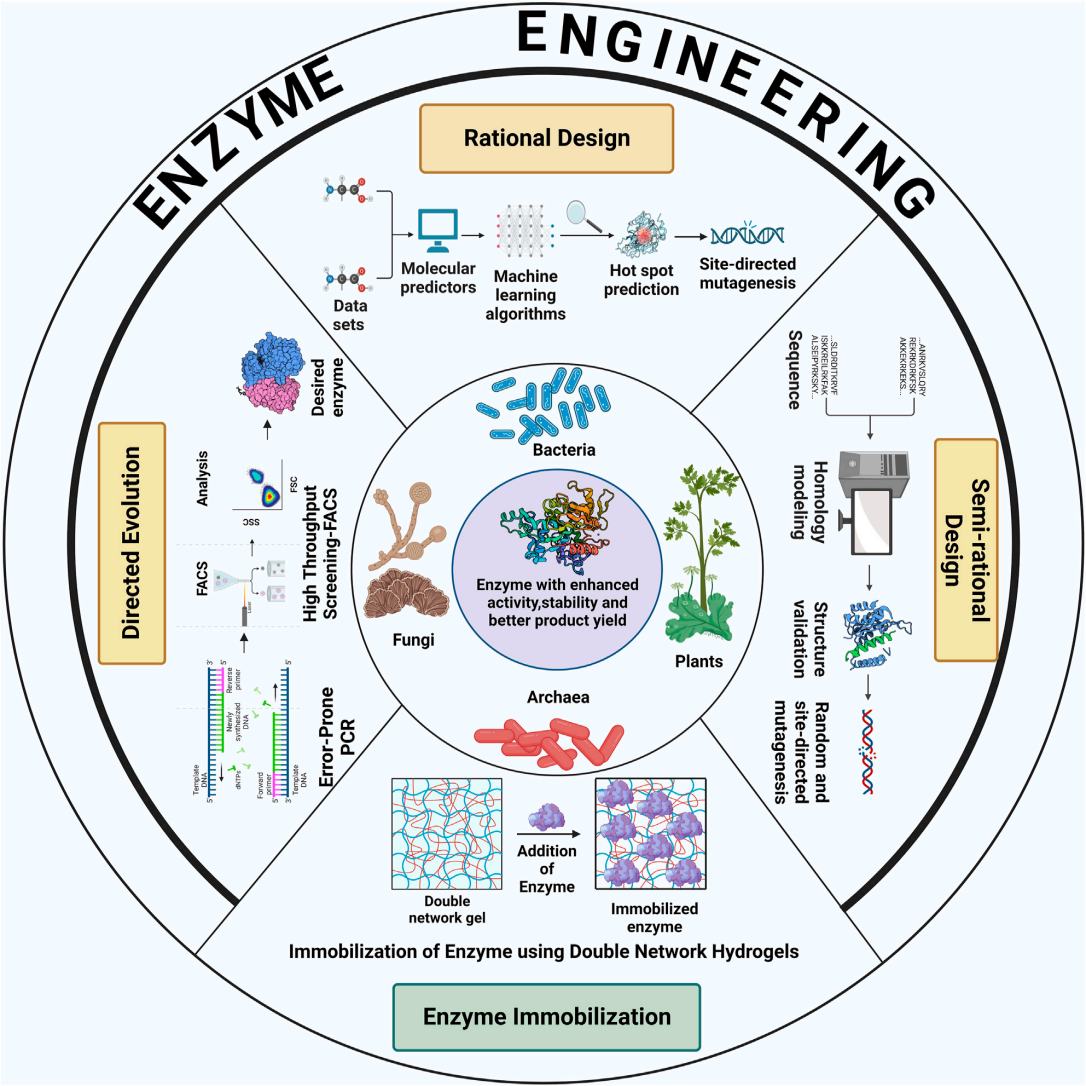
Schematic illustration of different strategies used for the improvement of catalytic properties and product yield of enzymes derived from various classes of extremophiles. The figure was created with BioRender.

Recent advances in the field of protein engineering have unlocked new pathways to employ several biotechnological techniques to meet the ever-increasing demands of industrial biocatalysis and biotransformations. Enzymes engineering require the implementation of different enzyme-designing strategies such as random mutagenesis, site-directed mutagenesis, enzyme immobilization, molecular modeling, DNA shuffling, and peptidomimetics. Broadly, the methodologies for enzyme engineering can be classified into three major groups: directed evolution, rational design and semi-rational design. Figure 1 schematically shows various strategies to enhance enzyme activity and stability for increased product yield.

#### 2.1.1 Directed evolution

The process of directed evolution adopts the idea behind natural selection in that it integrates random mutations in the gene encoding for the enzyme of interest (Zeymer & Hilvert, 2018). This results in the generation of DNA libraries, which are used to develop different variants of the enzyme, either *in-vitro* (in cells or free solution), or *in-vivo* (in living organisms). High throughput screening techniques are, then, exploited to select the most preferred enzyme variant based on its enhanced properties. Directed evolution has proven to be one of the most successful approaches of enzyme engineering for the production of enzymes with improved catalytic activity, thermostability, substrate-specificity and stereoselectivity. This method of protein engineering is often applied in cases where there is limited knowledge about the structure-function relationships within the enzyme (Laksmi et al., 2020).

In this approach, techniques such as error-prone polymerase chain reaction, staggered extension process and DNA shuffling are used to introduce mutations randomly in the genetic assembly encoding the protein of interest, generating large libraries of DNA (Bloom et al., 2005; Sharma et al., 2021). Screening these libraries to search for the enzyme variant with desirable attributes is the most challenging part of this method. Several analytical methods like chromatography, fluorescence or NMR spectroscopy to high throughput screening techniques such as fluorescence-activated cell sorting (FACS), microtiter-plate screening and *in-vitro* compartmentalization have been employed to facilitate the process of screening and selection. *In-vitro* selection systems like ribosome display or mRNA display and phage-assisted continuous evolution (PACE) are known to be potent tools to increase the rate of the evaluation of enzyme variants (Ali et al., 2020). Arnold and co-workers (K. Chen & Arnold, 1993) first experimentally implemented the Eigen concepts (Eigen & Gardiner, 1984) of directed evolution of enzymes. In this pioneering work, variant of subtilisin enzyme (used in detergent, cosmetic and food processing industry) was obtained from several sequential rounds of mutagenesis and screening which showed around 256 fold increased enzyme activity than wild-type enzyme (Arnold, 1993).

#### 2.1.2 Rational design

Rational design is one of the most widely used methods of enzyme engineering, which is based on the association of the structural aspects of an enzyme with its functions. This method requires prior knowledge about the sequence and structure of the enzyme of interest to identify potential amino acid residues within the enzyme that can be mutated, without disrupting the catalytic activity of the enzyme. Specific amino acid residues are either substituted, inserted or deleted by subjecting them to site-directed mutagenesis (Dadwal et al., 2020). In order to identify specific amino acids, the three-dimensional structure of the enzyme is analysed to extract information about the amino acid residues located within or close to the active site of the enzyme. These amino acids come in direct contact with the substrate and hence, are targeted to be mutated, as mutations in these sites would prove to be significant in altering the catalytic properties of the enzymes. Mutation hotspots can be identified by developing enzyme models using techniques like homology modelling and threading. This is achieved by using *in silico* tools like Rosetta (Mills et al., 2013), IntFOLD (Roche et al., 2011), I-TASSER (Yang et al., 2014), Swiss-Model (Waterhouse et al., 2018) and Protein Homology/AnalogY Recognition Engine (Kelley & Sternberg, 2009). Performing multiple sequence alignment to compare homologous protein sequences with the sequence of the enzyme of interest provides valuable information about the conserved sequences of amino acids in the enzyme (Sebestova et al., 2014). This is particularly useful because stretches of conserved amino acid residues are important for maintaining the core structure of the enzyme and therefore, they cannot be subjected to mutation. However, the amino acid residues which are less conserved could be mutated for enzyme engineering. The information about the interaction of the active site residues with its substrate can be generated through various computational applications like molecular dynamics (MD) simulation (Anbar et al., 2012) and molecular docking. These mutant enzymes are further screened to obtain the mutant with the most desirable properties, such as increased catalytic activity, specificity for non-natural substrate as well as multiple substrates, enantioselectivity, and solubility. Rational design approach was utilized in creating variants of TesA thioesterase enzyme in *Escherichia coli* in which iterative protein redesign and optimization (IPRO) method (Pantazes et al., 2015) was used. The enzyme variant improves medium-chain free fatty acid (substrate) specificity and maintain high thioesterase activity (Grisewood et al., 2017).

#### 2.1.3 Semi-rational design

Semi-rational design of enzyme engineering involves the use of a combination of approaches employed in both direct evolution and rational design to combat the disadvantages faced (like extensive screening process and unavailability of detailed structural information of enzymes) when these methods are deployed individually (Ali et al., 2020). In semi rational design, sequence and structure-based information about the concerned enzyme is considered to select promising target sites which could be altered by combining random mutations and site-directed mutations. Computational algorithms, further, help to predict and eliminate the possibility of unbeneficial mutations. This approach creates small libraries with functional content that assists the desired change in protein conformation. This strategy for engineering enzymes focuses on the effect of substitution of amino acids within the catalytic domain of the enzyme and has been found to be extremely successful in terms of designing new enzymes and enhancing the catalytic activity, stereoselectivity and stability of existing enzymes. The development of *in-vitro* experimental assays depending on the information pipeline is critical to modify existing proteins as well as biosynthesis of novel enzymes possessing properties that are beneficial to biocatalytic processes in the industry. Semi rational design generally adopts sequence, structure and random mutagenesis-based strategies. These strategies are heavily dependent on computational techniques.

Analysis of protein sequences, for identification of sites that might be mutated, can be done using computational tools like HotSpot Wizard server and 3DM database which compile information about the sequence data retrieved from different repositories (Pavelka et al., 2009; Steiner & Schwab, 2012). The amino acid sequences within the active site are directly associated with its catalytic properties. Various methods like multiple sequence analysis and construction of phylogenetic trees give useful insights into the conservation of homologous protein sequences and their evolutionary history. This allows easier selection of amino acid residues which can be substituted in the process of enzyme engineering. Identification of amino acid residues in the active site of enzymes facilitate the generation of small and smart libraries.

In structure-based semi-rational design, the structural information of enzymes is taken into account to enhance enzymatic activity (Siloto & Weselake, 2012). In the absence of crystal structure, models of enzymes can be produced using the information about its sequence by methods like homology modelling and threading. Computational evaluation of these models is carried out based on the energetics of amino acid mutations in the enzyme using the available rotamer libraries and backbone recognition (Ebert & Pelletier, 2017). Forcefield-based various algorithms like FoldX (Buß et al., 2018), TANGO (Musil et al., 2019), SNPEffect (De Baets et al., 2012) can score the enzyme mutants by calculating the difference in their free energies. *De novo* enzymes having specific catalytic properties can also be synthesized using computational methods. This approach is particularly useful to understand enzyme-substrate interactions (Kries et al., 2013). Virtual modulations in amino acid substitutions to generate desirable qualities in the novel enzyme screens out only a few designs for the *in-vitro* engineering of enzymes. Molecular dynamics simulation and quantum mechanical calculation predict the exact locations of amino acid residues within the active site of the enzyme. To achieve this, programmes such as RosettaMatch (Siegel et al., 2010), YASARA (Zorn et al., 2018) can be utilized.

In the absence of sequence and structural information of enzymes, random mutagenesis technique is employed. In this technique, large enzyme libraries are created by incorporating random mutations in the enzyme. Variants of enzymes with superior properties are screened out. Upon recognition of amino acid residues which are mutated in the superior variety of enzymes, site-directed mutagenesis can be performed to generate the desired enzyme variant. Both these methods are used in semi-rational design (Chica et al., 2005). With the help of this strategy (using a combination of rational design and saturation mutagenesis), Rui et al. (2004) successful modified epoxide hydrolases. The modified enzyme could accommodate wide range of substrate through beneficial residues within active site of the enzyme. The modified enzyme also enhanced the substrate degradation rate by 4 to 7 fold.

### 2.2 Enzyme immobilization as a complementary strategy for enhanced enzyme stability and product yield

For the commercialization of enzyme-based catalysis, it is essential that these biocatalysts have the ability to be recovered and reused, making the catalytic process cost-effective. Immobilization of enzyme is a technique that imparts excellent stability to the enzyme and allows it to react with the substrate while being restricted in a predefined area (Soleimani et al., 2012; Maghraby et al., 2023). The process of immobilization requires the application of various interdisciplinary fields including biocatalysis, material science, biophysics, protein chemistry, chemical engineering and molecular biology. Enzyme immobilization is an immensely profitable technology in industries where the use of biocatalysts bears a huge impact on their economic feasibility. This technique makes sure that the process is optimized to control the operational time of enzyme usability and increases the total turnover number. Enzyme immobilization can also be used to shift the reaction equilibrium when the enzyme is required to work in non-conventional conditions. This wholly depends on the optimization of the enzyme-support system which forms the most critical part of enzyme immobilization. Designing of a potent immobilization technique consists of steps like selection and characterization of the enzyme to be immobilized, identification of a suitable matrix that can successfully bind to the enzyme conferring stability without affecting its catalytic properties and optimization of the operational performance of the resulting enzyme-support system such that it increases the yield and activity of the enzyme keeping in mind the reaction criteria to be used (van Pelt et al., 2008; Bernal et al., 2018).

Enzyme immobilization can be achieved through covalent attachment where the support material contains compounds or groups such as glyoxyl, glutaraldehyde, vinyl sulfone that can covalently bind to the amine and thiol groups in the enzyme. This method provides stability of the enzymes under various inactivating conditions. Osuna et al. (2015) used chitosan-coated magnetic nanoparticles to immobilize *Aspergillus niger* lipase (Osuna et al., 2015). Glutaraldehyde and glycidol were used to form covalent linkages between the enzyme and the polymer. The immobilized enzyme showed enhanced stability to withstand changes in pH and temperature and retained 80% of its initial catalytic activity after 15 hydrolytic cycles. Another method of immobilization requires the induction of weak forces between the enzyme and the support material through adsorption. Recently, hydrophobic groups like octyl and phenyl moieties have been introduced on the surface of carriers to enable their adsorption on the hydrophobic patches of the enzyme. For instance, Rueda et al. (2015) compared the efficiency of octyl-agarose and octyl-glyoxyl agarose to immobilize lipases from *Candida antarctica* (form B), *Thermomyces lanuginosus* (TLL) or *Rhizomucor miehei* by using both hydrophobic adsorption and covalent bonding techniques (Rueda et al., 2015). This study demonstrated that the stability and the activity of the enzymes were enhanced in organic solvents and also, that these enzymes can be reused for 5 hydrolytic cycles without any considerable loss in activity when they were immobilized on heterofunctional support (octyl-glyoxyl agarose). Thus, both covalent attachment and adsorption can be implemented together to favour enzyme–support interactions and to achieve high enzyme stability.

Entrapment and crosslinking enzyme aggregates are other strategies of enzyme immobilization. Crosslinking enzymes with suitable matrices involves the formation of enzyme aggregates by precipitation of enzymes and then cross-linking them with the support material such that the biocatalyst thus formed is active and fully functional. For example, nitrile hydratase from a haloalkaliphilic actinobacterium was precipitated with ammonium sulfate and glutaraldehyde was used to crosslink the enzyme to make stable aggregates for the hydration of acrylonitrile (van Pelt et al., 2008). The entrapment of enzymes in porous matrices is a trusted strategy to stabilize them and retain their native structure. The enzyme remains trapped in the gel–like matrix which allows easy diffusion of the substrate and the product. Ca-alginate beads were used by Arruda and Vitolo to entrap invertase from *S. cerevisiae* for sucrose hydrolysis. The immobilized biocatalyst showed high stability at pH 4.6 and 30°C (Arruda & Vitolo, 1999).

### 2.3 Extremozymes and Enzyme engineering

Enzyme engineering to improve enzyme specificity, stability, stereoselectivity and/or catalysis of a wider range of substrates, can be applied to several industrial sectors. The following section focuses on extremozymes as examples of industrially significant biocatalysts.

Enzymes have been extensively used in the food and beverage industry to enhance flavour and nutritional value of the product. Xylanases and starch processing enzymes such as amylases, pullulanases and transglutaminases are widely used in the production of food additives. These enzymes are often used at high temperatures in the industry. Therefore, engineering such enzymes to increase their thermal stability enhances their efficiencies and the product yield. Pang et al. improved the thermostability and activity of a type II pullulanase from the thermophile, *Anoxybacillus sp.* WB42 by surficial residue replacement and disulphide bond introduction (Pang et al., 2020). Xylanases are also known to be used in the biofuel industry as they can degrade hemicellulose, a structural component of plant cell wall. A thermostable xylanase from *Geobacillus stearothermophilus* was used by Hegazy et al. to create xylose-tolerant mutants by random mutagenesis, which have high catalytic efficiencies (Hegazy et al., 2019). In this case, in the absence of three dimensional structure of xylanase-xylose complex, random mutagenesis of the whole xylanase gene was prepared by error-prone polymerase chain reaction creating a huge library. Screening of the library produces xylose-tolerant mutants with M116I, L131P, and L133V, mutation which are within the N-terminus of α-helix 3. In comparison with the wild-type, the best xylose-tolerant mutant showed 3.5 fold higher ki value and 3 fold better catalytic capability. Apart from xylanases, lipases and endoglucanases are also employed by the biofuel industry for the production of biodiesel.

In the pharmaceutical industry, enzymes with therapeutic potential such as ketoreductases and transaminases play a significant role in the synthesis of chiral compounds. Such enzymes are engineered to make them highly enantioselective. For example, with the help of quantum mechanical calculations and molecular dynamic simulations, enantioselective mutants of *Lactobacillus kefir* ketoreductase, generated by Noey et al. which worked more efficiently with 3-thiacyclopentanone than with 3-oxacyclopentanone (Noey et al., 2015). This MD based method finds correlation between the relative fraction of catalytically feasible poses for the enantiomeric reductions and the actual experimentally observed enantiomeric ratio. It is shown that certain mutations alter binding site geometry which in turns enlarge binding pocket to accommodate the larger sulphur atom and thus enhancing the *S*-selectivity with 3-thiacyclopentanone.

The surfactant industry uses a wide range of lipases and proteases as bio-detergents. Enzyme engineering strategies are applied to these enzymes to increase their stability and retain their activity in the formulations. For instance, enzyme variant of a thermophilic esterase from *Sulfolobus solfataricus* P2 was produced by Shang et al. using site directed mutagenesis. This mutant showed higher specific activity at pH 5.5 and 80°C (Shang et al., 2010). Lactonase is another enzyme from *S. solfataricus*, which is important for the detoxification of organophosphorus compounds (Jacquet et al., 2017). Use of enzymes is a promising method to carry out the targeted breakdown of hazardous chemicals into safer substances. A summary of some noteworthy industrially important enzymes from extremophiles has been presented in Table 1.

**TABLE 1:** List of extremozymes engineered for various industrial applications.

Enzyme	Source organism	Enzyme engineering strategy	Application	References
α-glucosidase	*Thermus thermophilus* TC11	Random and site-directed mutagenesis	Commercial production of oligosaccharides	Zhou et al. (2015)
α-amylase	*Klebsiella pneumoniae* (CCICC no. 10018)	Site-saturation mutagenesis	Food processing and Biosynthesis applications	Pan et al. (2020)
Laccase	*Thermus thermophilus* SG0.5JP17-16	Site directed mutagenesis	Production of paper and applications in food industry	Zhu et al. (2021)
Pullulanase	*Bacillus acidopullulyticus*	Site directed mutagenesis	Breakdown of starch for the production of high-glucose syrup	Chen et al. (2019)
Type II pullulunase	*Anoxybacillus* sp. WB 42	Site directed mutagenesis using overlap extension PCR	Application in food and chemical industries for starch debranching process	Pang et al. (2020)
Chitinase	*Paenibacillus pasadenesis* CS0611	Site directed mutagenesis	Production of chito-oligosaccharides	Xu et al. (2020)
Esterase	*Sulfolobus solfataricus* P2	Site directed mutagenesis by overlap PCR	Detergent and leather industry	Shang et al. (2010)
Lipase	*Bacillus* sp.	Site directed mutagenesis	Application in production industries	Chopra et al. (2018)
Endo-glucanase and β-1,4-glucosidase chimeric enzyme	*Clostridium thermocellum*	Site directed mutagenesis	Bioethanol and biofuel production	Nath et al. (2019)
Cyclodextrin glycosyltransferase	*Bacillus circulans* STB01	Site directed mutagenesis	Application in food and pharmaceutical industry	Li et al. (2016)
Endo-1,4-β-xylanase	*Thermoascus aurantiacus* CBMAI756	Site directed mutagenesis	Degradation of lignocellulose biomass in biofuel industry	de Souza et al. (2016)
Lipase	*Geobacillus stearothermophilus* T6	Site directed mutagenesis	Production of biodiesel by methanolysis	Dror et al. (2014)
Maltogenic amylases	*Thermus* sp. strain IM6501	Random mutagenesis	Emulsifiers and defoaming agents in starch industry	Kim et al. (2003)
β-1,4-endoglucanase	*Pyrococcus horikoshii*	Site directed mutagenesis	Application in textile industry	Kang et al. (2007)
Endo-glucanase	*Thermobifida fusca*	Site directed mutagenesis	Biofuel and biopolymer production	Zhou et al. (2004)
Chitinase A	*Vibrio carchariae*	Site directed mutagenesis by PCR	Application in the pharmaceutical industry for production of chito-oligosaccharides	Pantoom et al. (2008)
Endoglucanase	*Alicyclobacillus acidocaldarius*	DNA amplification by PCR using enzyme template	Degradation of plant biomass to produce biofuels	Younesi et al. (2016)
Subtilisin protease	*Bacillus gibsonii*	Site directed mutagenesis	Application in surfactant industry	Jakob et al. (2013)
Maltogenic amylases	*Thermus sp*	Site directed mutagenesis by mega-primer method	Application in starch and carbohydrate industry	Kim et al. (2005)
D-hydantoinase	*Bacillu stearothermophilus* SD1	Rational design by overlapping PCR	Production of optically pure D- and L- amino acids	Lee et al. (2009)
Lipase CALB	*Candida antarctica*	Iterative Saturation mutagenesis based Directed Evolution approach	Application in pharmaceutical industry	Wu et al. (2013)
Acyl-aminoacyl peptidase	*Aeropyrum pernix* K1	Site directed mutagenesis	Synthesis and resolution of enantio-pure drug precursors	Liu et al. (2011)
Prolidase	*Pyrococcus horikoshii*	Error-prone PCR mutagenesis using Random Mutagenesis kit	Detoxification of organophosphates	Theriot et al. (2011)
Maltogenic amylases	*Bacillus thermoalkalophilus* ET2	DNA shuffling for random mutagensis	Starch and carbohydrate industry	Tang et al. (2006)
Aminotransferase Vfat	*Vibrio fluvialis*	Site-specific mutagenesis	Synthesis of chiral amines and amino acids	Midelfort et al. (2013)
Lactonase	*Sulfolobus solfataricus*	Rational method using structure-based approach	Bioremediation of organophosphorus compounds	Jacquet et al. (2017)
Prolidase	*Pyrococcus furiosus*	Random mutagenesis	Detoxification of organophosphorus compounds	Theriot et al. (2010)

## 3 Nature-inspired biocatalysis, enzyme engineering and extremozymes: case studies from the plant world!

**FIGURE 2:**
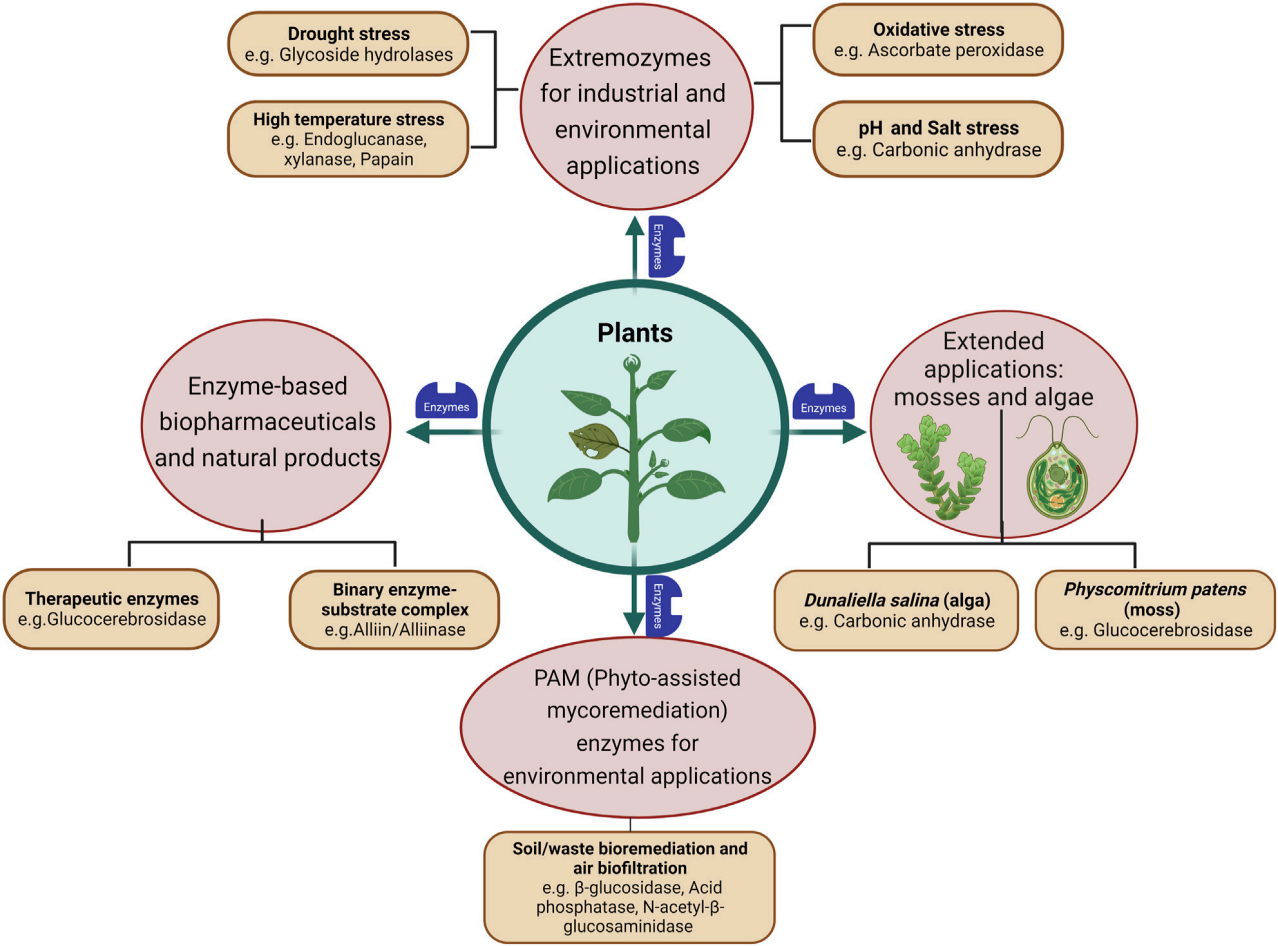
Schematic representation of plants as potential resources of enzymes (extremozymes) and related natural products. The figure was created with BioRender.

Extremozymes from bacteria and fungi, both natural and engineered ones, have been more popular for industrial applications like food, pharmaceuticals, biofuel, surfactant and bioremediation. However, the world of plants could also offer a distinct functional pool of novel biocatalysts, as there might be unique chemical reactions (metabolic pathways) that happen only in plants but not in microorganisms. The following sections highlight a few representative examples where extremozymes have been characterized and reported from plants. Specific plant enzymes, which could be selected in future for potential protein engineering experiments and a few which have been successfully engineered for improved biochemical characteristics like thermostability and substrate specificity, have also been discussed (Figure 2).

### 3.1 Thermostable glycoside hydrolases from (semi) arid plants

Glycoside hydrolases (GH; EC 3.2.1) are key enzymes of the CAZY (Carbohydrate active enzymes; http://afmb.cnrs-mrs.fr/CAZY/) category that are capable of hydrolyzing the glycosidic bonds between two carbohydrates or between a carbohydrate and a non-carbohydrate moiety. In plants, they participate in cell wall polymer rearrangements during plant growth (Calderan-Rodrigues et al., 2018), or facilitate plant defense/stress response via release of biologically active aglycones during insect infestation or pathogen/herbivore attack (Asati et al., 2021). They are often localized in the endoplasmic reticulum, plasma membrane, apoplast or plastids and come into contact with the corresponding glycosides (often stored in vacuoles) during cell damage. The GH superfamily includes 29 families of enzymes, such as cellulases, α- and β-glucosidases, β-galactosidases, β-xylosidases, α- and β-amylases, and lactases to name a few (Henrissat et al., 2001). Commercially, GHs find major applications in pulp and paper processing; textile manufacture; detergent, chemical and biofuel production; cosmetics industry; food industry (dairy, baking, juice and brewing) (Cobucci-Ponzano et al., 2011).

From an industrial perspective, deconstruction of plant biomass mainly requires depolymerizing starch, lignocellulose and pectin, that often require the use of enzyme cocktails (Lopes et al., 2018). Reduction in lignocellulose recalcitrance by thermal pre-treatment of plant biomass is considered a necessity even for enzyme-aided processes, therefore highlighting the importance of thermotolerant GHs. Mostly, bacterial and fungal enzymes have been used in the process, but plant enzymes present an equally attractive opportunity as these are naturally involved in cell wall turnover in plants. Whatever the source of enzyme, researchers have noted that natural enzymes may not be sufficiently active to enable economically viable bioprocesses, even at optimal conditions or might be subject to product inhibition (Chaudhari et al., 2023). Therefore, enzyme engineering becomes a necessity.

Starch is converted to fermentable sugars (with the help of α-glucosidase as one of the enzymes) during the industrial production of ethanol. This process requires temperature in the range of 65–73°C. Typical α-glucosidases (EC 3.2.1.20) are thermolabile at these temperatures, resulting in reduced efficiency of starch breakdown. (Mansfeld et al., 1997). A study reported on improving the thermostability of α-glucosidase from barley (Hordeumvulgare) by inserting proline residues (in place of threonine) at position 340 in the first turn of an α-helix. This kind of change has been suggested to stabilize a protein molecule. In this case, the thermostability (T50) of the mutant enzyme, T340P, was found to be 10°C higher than the non-mutant enzyme. Interestingly, the researchers got the clue for this threonine-to-proline substitution in barley by studying the relatively more thermostable α-glucosidases from three other plants, *viz.* sugarbeet (Beta vulgaris), spinach (Spinaceaoleracea) and Arabidopsis (*Arabidopsis thaliana*).

β-amylase (EC 3.2.1.2) is a maltose-producing exohydrolase of the starch degradation pathway and finds its application in brewing and baking industries. β-amylase from barley has been used for commercial production of maltose; however, its low thermostability has been a major challenge for industries. Recently, Ogundolie et al., 2022 reported on the isolation and characterization of a novel thermostable β-amylase from an underutilized climber legume, Diocleareflexa, and suggested it to be a potential candidate for further improvement via enzyme engineering (The enzyme was relatively stable for 140 min retaining 70% of its initial activity at 60°C and 67% of the activity at pH 6.0). Earlier, Daba et al., 2012 evaluated various solvent additives to enhance the activity and thermostability of wheat β-amylase. Solvent engineering is a relatively less employed method for rational control of catalytic activity for enzymes. Here the premise is that a combination of structural and environmental factors can be used to realize optimal enzyme activity, the latter requiring use of stabilizing additives in the solvent. It is supposed to enhance enzyme catalytic efficiency, ensure safety of the products and reduce the costs for production. The wheat β-amylase is extracted from wheat bran, considered an industrial byproduct. It was observed that modifications of the solvent with 182 mM glycine and 0.18% (w/v) gelatin increased the T50 by 5°C (Daba et al., 2012). It has been suggested that the mechanism for thermostabilization by additives could be due to reduction in the degree of water-solvation or deformation of a water shell around the protein.

It has been reported that the desert legumes such as *Prosopis cineraria* and *Cyamopsis tetragonoloba* harbor thermostable β-glucosidase enzymes (EC 3.2.1.21), capable of showing significant catalytic activity even above 50°C temperature (V Asati & Sharma, 2018; Asati and Sharma, 2019). These enzymes were specific towards isoflavone glycosides and did not show any activity with glycosides of other flavonoid categories, possibly due to difference in structure of the substrate, that is, flavonoid parent skeleton (benzene ring connected to Carbon 3 of C-ring in case of isoflavonoids, as against Carbon 2 in case of flavonoids). The plant β-glucosidases score over the bacterial counterparts by being relatively more tolerant to glucose inhibition (Monteiro et al., 2020). These traits make such enzymes (from plants in the arid regions of the world) potential candidates for protein engineering for further improvement in physiochemical characteristics, towards application in nutraceutical and food industries.

### 3.2 Digestion of lignocellulosic biomass by recombinant hyperthermophilic endoglucanase (EC 3.2.1.6) and xylanase (EC 3.2.1.8) enzymes

Lignocellulose biomass is primarily derived from plant materials, which includes waste from the agricultural and timber industries, forest residue, and municipal waste (Yousuf et al., 2020). It is a complex matrix mainly composed of lignin (15%–20%) and polysaccharides, such as cellulose (35%–50%) and hemicellulose (20%–25%), which are found in plant secondary cell walls (H. Chen, 2014). These polysaccharides can be broken down into monosaccharides, which can then be used to produce fermentable sugars. Lignocellulose is a valuable source for producing bioethanol and biogas, which are sustainable alternatives to petrochemicals and have gained increasing attention in recent years (Arevalo-Gallegos et al., 2017; Okolie et al., 2021). Additionally, lignocellulose offers advantages over other feedstocks for biofuel production, such as its great abundance, sustainable supply, lower direct food *versus* fuel competition, and fewer greenhouse gas emissions (Nanda et al., 2015).

However, the efficient conversion of lignocellulose to biofuel is a significant challenge in the production process, primarily due to the high processing costs involved in its pretreatment and enzymatic hydrolysis (Bhatia et al., 2020; Zhao et al., 2022). To reduce these costs, scientists have expressed lignocellulosic digesting enzymes in plants, but this approach also causes performance challenges, such as auto-hydrolysis of cell walls, stunted plant growth, poor yield, and stability of enzymes in extreme conditions such as high temperatures, extreme pH values, or high salt concentrations, which are required for the biomass processing steps (Marriott et al., 2016). Therefore, while this approach shows promise in reducing cost, another strategy has been to use recombinant hyperthermophilic lignocellulose digesting enzymes (glucanase and xylanase) in Arabidopsis plants which can withstand high temperatures (Mir et al., 2014; 2017). The endoglucanase and xylanase retain their hydrolyzing activity at around 100°C and around 80°C degrees, respectively (Graham et al., 2011; Verma et al., 2013). These enzymes were chosen because of their ability to withstand high temperatures and high pH during the lignocellulose treatment process, thereby improving biofuel yield.

### 3.3 Ascorbate peroxidase (EC 1.11.1.11) - Temperature induced isoforms in plants and structural clues

Plants are regularly exposed to various environmental stresses, including biotic and abiotic stress, which poses a significant challenge to their growth and survival (Suzuki et al., 2014). One consequence of such stresses is the notable increase in the production of reactive oxygen species (ROS), which are highly reactive molecules that can be either free radical or non-radical molecules. ROS are a natural by-product of plant cellular metabolism in association with oxidases, but when ROS levels go unchecked, it can be harmful to cells (F. K. Choudhury et al., 2017). To maintain redox homeostasis, the plants antioxidant defence system is activated, which includes several oxidases such as catalases (EC 1.11.1.21), ascorbate peroxidases, glutathione peroxidase (EC 1.11.1.9), superoxide dismutase (EC 1.15.1.1), glutathione S-transferase (EC 2.5.1.18), and glutathione reductase (EC 1.8.1.7), that regulate ROS levels (Gill & Tuteja, 2010; Trchounian et al., 2016)

Among these, ascorbate peroxidase (APX) is a crucial scavenger of free radicals and belongs to a family of heme-containing peroxidases that consists of four different isoforms based on their sub-cellular localization (Menossi Menossi et al., 2017). The central hydrogen peroxide scavenging system is present in chloroplasts, where APX exhibits a high affinity for H_2_O_2_ and catalyzes its conversion to H_2_O, using ascorbic acid as an electron donor (Smirnoff & Wheeler, 2000). By performing this function, APX protects plant cells from oxidative stress and stabilizes the internal biochemical state of the cell, leading to acclimatization as well as tolerance to various abiotic stresses (Das & Roychoudhury, 2014; Huang et al., 2019). However, APX also plays a role in the cytosol, mitochondria, and peroxisomes. Cytosolic APX isoforms are more sensitive to ascorbate reduction than stromal and thylakoid membrane-bound APX, which are of chloroplastic origin (Huang et al., 2019).

Under extremely low or high temperatures, various APX isoforms have been found to be induced in plants, which can enhance the plant’s ability to tolerate stress. For instance, cytosolic APX has been upregulated in potato tubers (Kawakami et al., 2002), maize (Tiwari & Yadav, 2020; Ramazan et al., 2021), and rice (Sato et al., 2001), while mitochondrial APX is expressed in Arabidopsis (Chew et al., 2003), and thylakoidal APX is expressed in tobacco plants (Sun et al., 2010). The differential expression of these various subcellular isoforms in regulating how plants adapt to temperature stress could provide insights at both the cellular and molecular levels, for their utilization in genetic engineering and other applications.

### 3.4 Carbonic anhydrases (EC 4.2.1.1): Role of unicellular green algae in pH- and salt-stress

Carbonic anhydrase is a type of metalloenzyme that is widely distributed across the biological kingdom (Chegwidden & Carter, 2000). Found in bacteria, plants, algae, and animals, including humans, and is known to function in a broad range of environmental stresses, including heat stress, CO_2_ stress, and salt stress, drought stress (Polishchuk, 2021). It plays a vital role in converting carbon dioxide to bicarbonate, along with a proton, in a reversible manner to carry out necessary biological processes such as carbon fixation, pH regulation, and ion transport (Imtaiyaz Hassan et al., 2013).

Plants produce reactive oxygen species (ROS) in response to stress conditions. Carbonic anhydrase is a crucial enzyme that helps to maintain the pH of the cell, thereby reducing the adverse effect of ROS in cellular damage (Di Fiore et al., 2018). In conditions, where CO_2_ is limited, bicarbonate can serve as a source of carbon for photosynthesis (Momayyezi et al., 2020). Additionally, carbonic anhydrase activity is upregulated during drought conditions, as it supports the plants in maintaining their water balance and protects them from dehydration by reducing water loss through the closure of stomata.

Carbonic anhydrase activity has also been reported in the halophilic algal species *Dunaliella salina*, a green alga that can survive in high salt concentrations where the solubility of CO_2_ is limited. To cope with these extreme conditions, *D. salina* increases the expression of carbonic anhydrase, located on the plasma membrane, to acquire CO_2_. This CO_2_ can then be assimilated for glycerol synthesis, which is an essential product of photosynthesis in halophiles (Jeon et al., 2016). It is also used in coupling the sequestered CO_2_ (from greenhouse gas emissions) into valuable products that include biofuels and bioplastics. For this several bacterial and algal sources of carbonic anhydrase are being reported (Y. Chen et al., 2020; Kumar et al., 2018). Thus, extremozymic forms of carbonic anhydrase can be explored for environmental hazard mitigation as well as sustainable production of industrially important products.

### 3.5 Papain—An industrially important plant protease with enhanced thermostability by protein engineering technique

Among industrially relevant enzymes, proteases represent more than 60% of the enzyme market share (Fernández-Lucas et al., 2017). Proteolytic enzymes from plants, particularly the cysteine proteases, have attracted significant commercial interest, due to their wide substrate specificity and catalytic activity over broad pH and temperature ranges (Mamboya & Amri, 2012). These enzymes have found their applications in medicine (e.g., synthesis of bioactive peptides and treatment of burn wounds) as well as food (e.g., preparation of protein hydrolysates, removal of food allergens and tenderization of meat) and detergent industry (hydrolytic degradation of difficult protein stains) (Sharma et al., 2021). A few other novel applications include antimicrobial food packaging, dental caries removal agent, tooth-whitening additive in toothpastes and skin-care products (Cynthya et al., 2014; Epple et al., 2019).

Papain (EC 3.4.22.2) is a cysteine endopeptidase isolated from latex of the tropical plant Carica papaya. Physiologically, the enzyme is involved in plant growth, development, senescence and programmed cell death (Martínez et al., 2007). As per Market Research Future (Market Research Future (2017) *Global Meat Tenderizing Agents Market Research Report- Forecast to 2023. ID: MRFR/F-B & N/2125-HCRR*, 2017), owing to its broad protein substrate specificity and better structural/functional integrity in different operational conditions, papain outsold other plant-derived proteases like bromelain (from Ananascomosus) and ficin (from Ficuscarica). It has also been considered less expensive than microbially sourced proteases (Dahiya et al., 2001).

Papain structure has two domains, L and R, with its catalytic cleft located at the interface of the domains. Choudhury et al., 2010 improved thermostability of papain through structure-based protein engineering. As per the authors, in the major protein engineering approaches (random mutagenesis followed by selection, rational design based on 3D structure of protein, and consensus approaches using statistics and sequence databases), a trade-off occurs between the rigidity required for stability and flexibility required for activity. To take care of this aspect, they produced a triple mutant by substituting three amino acids in the interdomain region of papain (Val32, Gly36 and Lys174 in the interdomain region were mutated to Ser, Ser and Arg, respectively). The researchers ensured that the chosen positions for substitution were away from the catalytic region (thereby not affecting enzyme activity). The change resulted in multiple hydrogen bonds and salt bridges, thereby improving the thermostability (the half-life extended by 94 min at 60°C and 45 min at 65°C compared to the wild type).

Generally, the activity and specificity of an enzyme are altered by introducing mutations at the catalytic and substrate-binding sites. A different approach was suggested by Dutta et al., 2016 who carried out a single amino acid substitution in the pro-peptide of papain to modify its substrate specificity. The pro-peptide is a part of the protease that functions as an intra-molecular chaperone (that catalyzes the folding of the catalytic domain) as well as an inhibitor of the protease (that blocks the active site cleft in the zymogen form of the enzyme). Crystallographic studies of the mutant proenzymes demonstrated that no gross conformational changes had occurred in the catalytic cleft. One of the mutants, I86L, showed 29.1 times higher kcat/Km values (against protein substrates having Leu at P2 position) compared to the wild type enzyme.

## 4 Other special examples of nature-inspired biocatalysis

### 4.1 Plant-derived enzymes as biopharmaceuticals: an emerging avenue

The historic achievement of producing a therapeutic enzyme from a genetically engineered carrot cell line and its US-FDA approval in 2012, points to plants as an emerging source of enzymes, in addition to other valuable bioactive (Meyer & Schmidhalter, 2014; Najmi et al., 2022).

Gaucher’s disease is a glycolipid storage disorder, where enzyme replacement therapy (ERT) is the standard treatment. Previously the production of the (imiglucerase) cerebrosidase enzyme had several challenges, the important factor being its restricted supply owing to viral contamination in production units (Hollak et al., 2010). Subsequently, the development of taliglucerasealfa, a recombinant human β-glucocerebrosidase (EC 3.2.1.45) produced in carrot cells (Fox, 2012), with the same protein sequence as imiglucerase, highlights the importance of plant cell culture platform in the biopharmaceutical industry (Zimran et al., 2012). Plant cell culture is a promising technology for sustainable production of high-value plant-derived products, as it is not affected by seasonal variations and does not require extensive farming or agricultural investments. Additionally, it presents a reduced risk of viral contamination and improved long-term safety compared to mammalian cell bioreactors (Caretto et al., 2010; Ochoa-Villarreal et al., 2016).

The success with plants extends to the mosses (bryophytes). Mosses often have the advantages of being a simple model system for not only understanding complex plant biosystems but also being amenable to scale-up production in bioreactors and for genetic engineering.

*Physcomitrium patens*, a type of bryophyte, has shown promising results in optimizing the glycan structure for producing alpha-glucocerebrosidase and beta-glucocerebrosidase, which are used to treat Fabry (Shen et al., 2016) and Gaucher’s diseases (Reski et al., 2015). Compared to other cell culture systems, *Physcomitrium patens* has demonstrated increased stability, no cytotoxicity and improved pharmacokinetics, making it a promising candidate for developing safe and effective therapies. Moreover, the use, *P. patens* may not be limited to lysosomal storage diseases and could be explored for treating other medical conditions as well (Campos et al., 2020).

### 4.2 Enzyme-substrate complexes from the plant world for therapeutic applications

The remarkable understanding of utilizing enzyme-substrate complexes for therapeutic purposes provides yet another inspiration from the plant world for the industrial production of enzymes. The rapid conversion of an inactive precursor to an active form observed in plants was the premise for exploring anti-cancer and anti-microbial effects of binary systems of enzyme and its substrate. Phytoanticpins are secondary metabolites produced by plants, either naturally or in response to pathogen attack. In the presence of an enzyme β-glucosidases (EC 3.2.1.21), these compounds are activated and have been shown to promote health benefits through a binary system involving enzyme-substrate complexes (Vinther et al., 2008; Ullrich et al., 2019). The report of a binary system in garlic, where an allin/alliinase combination was used as a substrate/enzyme combination, showed antifungal activity (Fry et al., 2005). The glucosinolate/myrosinase system is another binary combination from mustard that increased *Lactobacillus* population during fermentation (Wang et al., 2021). Additionally, these substrate-enzyme complexes have potential to provide a broad spectrum of targets, possibly by multiple substrate-enzyme formulations (Estevam et al., 2015). Such substrate-enzyme complexes provide an alternative approach to the use of either enzyme only, or enzyme inhibitors/activators, for pharmacological uses.

### 4.3 Extracellular enzymes of fungal isolates from plants: biotechnological applications

Several plants have therapeutic potential for the treatment of various diseases. However, one of the challenges is the fungal contamination of plants, which can pose problems and risks to plant health and its natural products (Yun et al., 2013). Interestingly, a study of the extracellular enzyme profile in xerophilic fungi growing as a contaminant over dried medicinal plants, revealed that these fungal isolates (such as *A. fumigatus and A. nidulans*) from medicinal plants have the ability to degrade hydrocarbons. The hydrolase activity of these fungi, such as β-glucosidase (EC 3.2.1.21) activity, was found to be the highest, followed by activities of other enzymes, namely, acid phosphatase (EC 3.1.3.2), N-acetyl-β-glycosaminidase (EC 3.2.1.30), and naphthol-AS-BI-phosphohydrolase. Although fungal contamination over plants is known to produce mycotoxins, there may be such instances of useful extracellular enzymes produced which can be used in industrial applications, as well as for combating environmental challenges, through air biofiltration and bioremediation (plant-assisted mycoremediation, PAM) of soil and wastes (Janda & Ulfig, 2009). Such fungi growing on medicinal plants also display the ability to act on a variety of substrates, which again makes them an attractive biotechnological strategy for industrial and environmental applications (Van Zyl et al., 2010).

Such fungal enzymes are also reported from non-medicinal crops such as maize as part of plant assisted mycoremediation (PAM) approach. It was shown that the organic volatile environmental pollutant, polycyclic aromatic hydrocarbons (PAH), was effectively remediated by maize-assisted mycoremediation through increased fungal biomass, microbial as well as manganese peroxidase enzyme activity, thereby suggesting PAM approach to be suitable for *in situ* soil remediation applications (Košnář et al., 2019).

## 5 Conclusion and future prospective

Enzymes are now established as the green catalysts preferred for industrial productions and environmental remediation. Biotechnological advances are providing further improvisations in the enzyme design, kinetics and the overall productivity. The naturally occurring category of extremozymes that inherently withstand extremes of conditions such as temperature, pH, salinity, and other stresses have found immense applications in industrial settings as well as harsh environments for bioremediation applications. While microbes have been widely reported for sourcing of extremozymes, this review presents the largely untapped potential of extremophilic plants as complementary sources of extremozymes. Such plant enzymes offer valuable structural insights for developing stable and robust enzymes, for instance thermostable proteases based on papain. Plant cell culture is yet another avenue to increase the yield of enzymes, without disturbing the ecosystem (that is, no felling of trees and minimal use of solvents for extraction of plant-based products). In the present review, we have also brought out some exotic instances of plant based biocatalysis, ranging from algae and mosses to higher plants. The plant fungal interaction which is generally construed as a harmful host pathogen interaction, is now finding some useful applications such as, PAM outlined in the above section. To conclude, plants literally and scientifically represent the green avenue for nature inspired biocatalysis.
